# FKBP65-dependent peptidyl-prolyl isomerase activity potentiates the lysyl hydroxylase 2-driven collagen cross-link switch

**DOI:** 10.1038/srep46021

**Published:** 2017-04-05

**Authors:** Yulong Chen, Masahiko Terajima, Priyam Banerjee, Houfu Guo, Xin Liu, Jiang Yu, Mitsuo Yamauchi, Jonathan M. Kurie

**Affiliations:** 1 Department of Thoracic/Head and Neck Medical Oncology, The University of Texas MD Anderson Cancer Center, Houston, TX-77030, USA; 2 Oral and Craniofacial Health Sciences, School of Dentistry, University of North Carolina at Chapel Hill, Chapel Hill, NC-27599, USA

## Abstract

Bruck Syndrome is a connective tissue disease associated with inactivating mutations in lysyl hydroxylase 2 (LH2/PLOD2) or FK506 binding protein 65 (FKBP65/FKBP10). However, the functional relationship between LH2 and FKBP65 remains unclear. Here, we postulated that peptidyl prolyl isomerase (PPIase) activity of FKBP65 positively modulates LH2 enzymatic activity and is critical for the formation of hydroxylysine-aldehyde derived intermolecular collagen cross-links (HLCCs). To test this hypothesis, we analyzed collagen cross-links in *Fkbp10*-null and –wild-type murine embryonic fibroblasts. Although LH2 protein levels did not change, FKBP65 deficiency significantly diminished HLCCs and increased the non-hydroxylated lysine-aldehyde–derived collagen cross-links (LCCs), a pattern consistent with loss of LH2 enzymatic activity. The HLCC-to-LCC ratio was rescued in FKBP65-deficient murine embryonic fibroblasts by reconstitution with wild-type but not mutant FKBP65 that lacks intact PPIase domains. Findings from co-immunoprecipitation, protein-fragment complementation, and co-immunofluorescence assays showed that LH2 and FKBP65 are part of a common protein complex. We conclude that FKBP65 regulates LH2-mediated collagen cross-linking. Because LH2 promotes fibrosis and cancer metastasis, our findings suggest that pharmacologic strategies to target FKBP65 and LH2 may have complementary therapeutic activities.

Covalent intermolecular cross-linking of collagen is critical for the stability of connective tissues. It is initiated by the conversion of the lysine (Lys) and hydroxylysine (Hyl) residues in the N- and C-terminal telopeptides of collagen to aldehydes (Lys^ald^ and Hyl^ald^, respectively) by the action of lysyl oxidases (LOXs)^1^. These reactive residues can then form an iminium bond to a vicinal ε-amino group of Lys or Hyl on the neighboring molecules. Cross-linking between Hyl^ald^ and a juxtaposed Hyl or Lys residue leads to the formation of the divalent cross-links dehydrodihydroxylysinonorleucine (deH-DHLNL) and deH-hydroxylysinonorleucine (deH-HLNL), which spontaneously rearrange to form stable ketoamines that then mature into the trivalent cross-links, pyridinoline (Pyr) and deoxy-Pyr (d-Pyr) (Supplementary Fig. 1). Most of the non-hydroxylated Lys^ald^-derived cross-links, including deH-hydroxylysinonorleucine (deH-HLNL), which results from a cross-link between Lys^ald^ and Hyl, and deH-histidinohydroxymerodesmosine (deH-HHMD), which results from cross-linking between one Hyl, one His, and two Lys^ald^ residues (Supplementary Fig. 1), are structurally unstable^2^,^3^,^4^. Thus, the state of Lys hydroxylation in the telopeptides is a critical determinant of collagen stability.

Lys hydroxylation of collagen takes place in the endoplasmic reticulum (ER), and the reaction is catalyzed by three isoforms of lysyl hydroxylases (LH1-3)^5^. Of these LHs, LH2 is the only LH that is capable of hydroxylating Lys residues in the telopeptides^6^,^7^,^8^,^9^ and is thus a key driver of collagen cross-link stability. The biological importance of LH2 is evidenced by its critical involvement in various pathologies such as Bruck syndrome, fibrosis, and cancer metastasis^7^,^10^,^11^,^12^,^13^. Bruck syndrome is a rare autosomal recessive disorder characterized as osteogenesis imperfecta with congenital joint contractures^14^,^15^,^16^. Bruck Syndrome is further classified into two types, types 1 and 2. They are phenotypically indistinguishable but caused by mutations in two distinct genes, i.e. *FKBP*10 and *PLOD*2 (encoding LH2), respectively^10^,^17^,^18^. The former gene encodes FK506 binding protein 65 (FKBP65, hereafter), an endoplasmic reticulum-resident peptidyl prolyl *cis-trans* isomerase (PPIase) and chaperone molecule. The common molecular/biochemical phenotype in Bruck Syndrome types 1 and 2 is the abnormal collagen cross-linking pattern in bone type 1 collagen caused by defective Lys hydroxylation in the telopeptides^19^. *Fkbp10*-null mice, which have abnormal internal rotation of arms without contractures and die during embryogenesis, have reduced collagen telopeptide Hyl levels^20^. Very recently, Gjaltema *et al*. reported that FKBP65 forms a complex with LH2, but not LH1 or LH3, and promotes LH2 dimerization, which is crucial for LH2 enzymatic activity^21^. However, the molecular mechanism by which FKBP65 regulates LH2 activity and its functional outcomes, i.e. cross-linking, are still not clear. In this study, we addressed if FKBP65-dependent peptidyl-prolyl isomerase domain/activity is responsible for its interaction with LH2 and ultimately to regulate the formation of LH2-mediated collagen cross-links.

## Results

### FKBP65-dependent PPIase activity potentiates the LH2-mediated collagen cross-link switch

**Figure 1:**
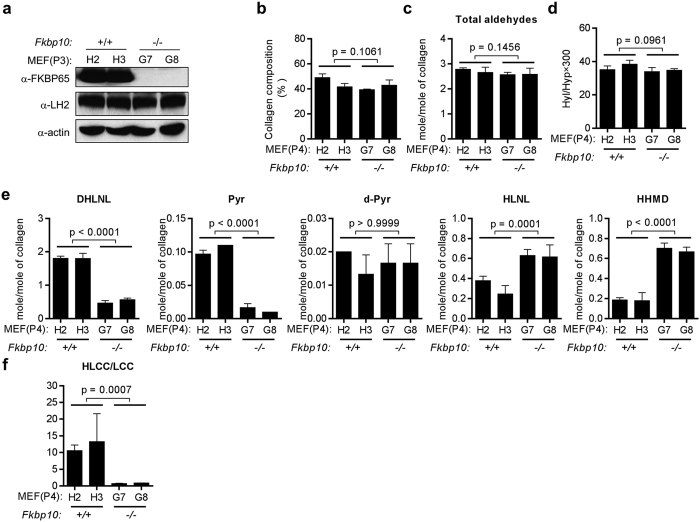
Collagen cross-linking analysis of *Fkbp10*^+/+^ and *Fkbp10*^−/−^ MEFs. (**a**) The blots show the results of Western blot analysis of the MEFs. Actin was used as the loading control. (**b–f**) Quantification of collagen composition (**b**); total aldehydes (**c**); Hyl/Hyp × 300 (**d**); HLCCs (DHLNL, Pyr, d-Pyr), LCCs (HHMD), and HLNL (**e**); and the ratio of HLCCs/LCCs (**f**) in the MEFs (passage 4). The total aldehyde values were the sum of DHLNL, HLNL, 2 × Pyr, 2 × d-Pyr, and 2 × HHMD. The HLCC/LCC ratio was calculated as (DHLNL + Pyr + d-Pyr)/HHMD. Data are the means ± SDs of triplicate samples. P values, two-tailed Student t-test.

To determine whether FKBP65 regulates the formation of LH2-mediated collagen cross-links, we quantified collagen cross-links in the extracellular matrices produced by congenic MEFs that are *Fkbp10*^*+/+*^ (H2 and H3) or *Fkbp10*^−/−^ (G7 and G8) (Fig. 1a). The collagen content per total proteins in the matrices was similar between *Fkbp10*^−/−^ and *Fkbp10*^+/+^ MEFs (Fig. 1b), suggesting that the absence of FKBP65 in MEFs did not significantly affect collagen protein synthesis (Fig. 1b). Total aldehydes involved in the cross-links did not change in *Fkbp10*^−/−^ MEFs (Fig. 1c), indicating that LOX activity was not affected. The major effect of the FKBP65 deficiency on collagen cross-linking was a switch from the Hyl^ald^- to the Lys^ald^-derived pathway (see below). The extent of Lys hydroxylation in collagen was similar between the *Fkbp10*^+/+^ and *Fkbp10*^−/−^ MEFs (Fig. 1d), suggesting that loss of FKBP65 does not significantly affect hydroxylation of Lys residues in the helical domains of collagen, as the majority of Hyl residues are in the helical domain. The two most abundant HLCCs, DHLNL and Pyr, were present at markedly lower levels in *Fkbp10*^−/−^ MEFs (less than 30% for DHLNL and 13% for Pyr of *Fkbp10*^+/+^). The least abundant HLCC, d-Pyr, was present at similar concentrations (Fig. 1e). Conversely, the major LCC, HHMD and likely HLNL (see below), were present at significantly higher concentrations in *Fkbp10*^−/−^ MEFs (Fig. 1e). Consequently, the HLCC-to-LCC ratio was markedly lower in *Fkbp10*^−/−^ MEFs (Fig. 1f). It should be noted that HLNL can be classified as an LCC or HLCC depending on its derivation and was thus excluded in this calculation^4^. However, an increase in HLNL in *Fkbp10*^−/−^ MEFs was due likely to the switch from Hyl^ald^ to Lys^ald^ in the telopeptides as the helical Lys hydroxylation was almost identical (see above), and changes in other cross-links, i.e. higher LCC and lower HLCCs, also support this notion. LH2 protein levels were similar in *Fkbp10*^−/−^ and *Fkbp10*^+/+^ MEFs (Fig. 1a). Thus, FKBP65 deficiency caused markedly diminished LH2-mediated collagen cross-links without affecting LH2 gene expression and protein levels.

**Figure 2:**
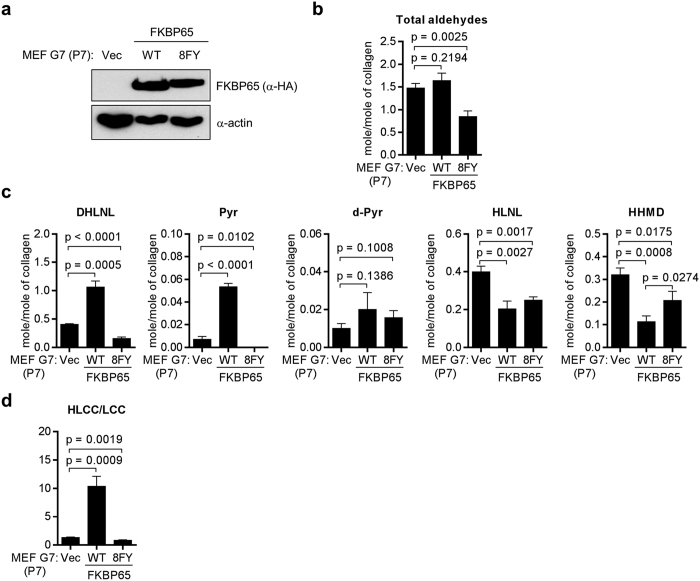
FKBP65 regulates LH2-induced collagen cross-linking through its PPIase activity. (**a**) Western blot analysis of *Fkbp10*^−/−^ G7 MEFs (passage 7) stably transfected with vectors expressing vector control (Vec), HA-tagged WT FKBP65, or isomerase-dead mutant (8FY) FKBP65. Actin was used as the loading control. (**b–d**) Quantification of total aldehydes (**b**); HLCCs (DHLNL, Pyr, d-Pyr), LCCs (HHMD), and HLNL (**c**); and the ratio of HLCCs/LCCs (**d**) in the *Fkbp10*^−/−^ G7 MEF transfectants. The total aldehyde values were the sum of DHLNL, HLNL, 2 × Pyr, 2 × d-Pyr, and 2 × HHMD. The HLCC/LCC ratio was calculated as (DHLNL + Pyr + d-Pyr)/HHMD. Data are the means ± SDs of triplicate samples. P values, two-tailed Student t-test.

To determine whether LH2-mediated collagen cross-linking requires FKBP65’s PPIase activity, we reconstituted *Fkbp10*^−/−^ MEFs with wild-type FKBP65 or a mutant FKBP65 (8FY) protein that lacks isomerase activity by mutating the residues (F79Y, F142Y, F191Y, F254Y, F303Y, F366Y, F417Y, and F478Y) corresponding to the residues Phe-67 and Phe-130 of FKBP52 in all four PPIase domains on the basis of evidence that Phe-67 and Phe-130 are required for isomerase activity of FKBP52^22^ (Fig. 2a). Relative to empty vector-transfected controls, *Fkbp10*^−/−^ MEFs reconstituted with wild-type FKBP65 did not affect the total aldehydes involved in the cross-links analyzed (Fig. 2b). Relative to *Fkbp10*^−/−^ MEFs transfected with empty vector, those transfected with wild-type FKBP65 had increases in HLCCs (DHLNL, Pyr, and d-Pyr) (Fig. 2c) and decreases in HLNL and the LCC HHMD; HHMD levels were 36% lower in FKBP65-reconstituted than empty vector-transfected *Fkbp10*^−/−^ MEFs (Fig. 2c). Consequently, reconstitution with WT FKBP65 increased the HLCC-to-LCC ratio by more than 7-fold (Fig. 2d). In contrast, reconstitution with the PPIase-inactive FKBP65 mutant led to a slight decrease in total aldehyde levels (Fig. 2b) and no increase in the levels of HLCCs or the HLCC-to-LCC ratio (Fig. 2c,d). Thus, FKBP65’s PPIase activity was required to generate LH2-mediated collagen cross-links.

### FKBP65 forms a complex with LH2 through its PPIase domains

**Figure 3:**
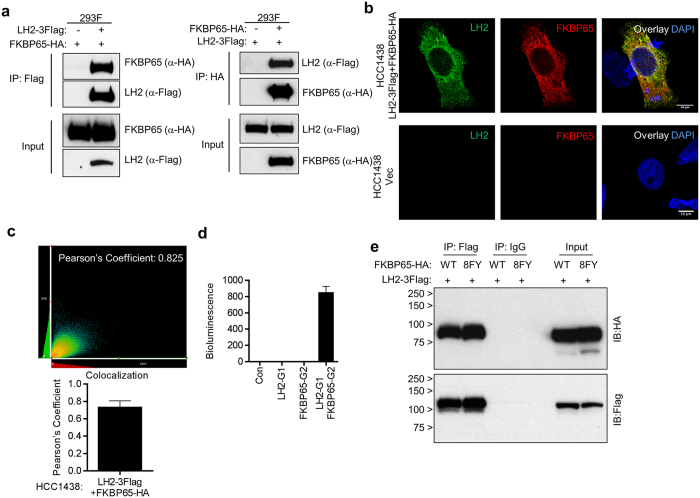
FKBP65 forms a complex with LH2. (**a**) Western blot analysis of 293F cells transfected with the indicated plasmids. LH2 and FKBP65 were immunoprecipitated with anti-Flag or -HA antibodies, respectively, and the immunoprecipitates were subjected to Western blot analysis. (**b**) Representative photomicrographs of immunofluorescence staining of HCC1438 cells co-transfected with LH2-3Flag and FKBP65-HA expression vectors. (**c**) (Top panel) Representative cytofluorogram showing the overlapping pixel signals from green (LH2) and red (FKBP65) channels. (Bottom panel) Bar graph showing the Pearson coefficient of colocalization values from 40 cells (mean ± SD). (**d**) Luciferase activities in total cell lysates of 293F cells transfected with the indicated plasmids. Data in the bar graph are means ± SDs of triplicate samples. (**e**) Western blot analysis of 293 F cells transfected with the indicated plasmids for 2 days. LH2 was immunoprecipitated with anti-Flag antibody, and the immunoprecipitates were subjected to Western blot analysis.

**Figure 4:**
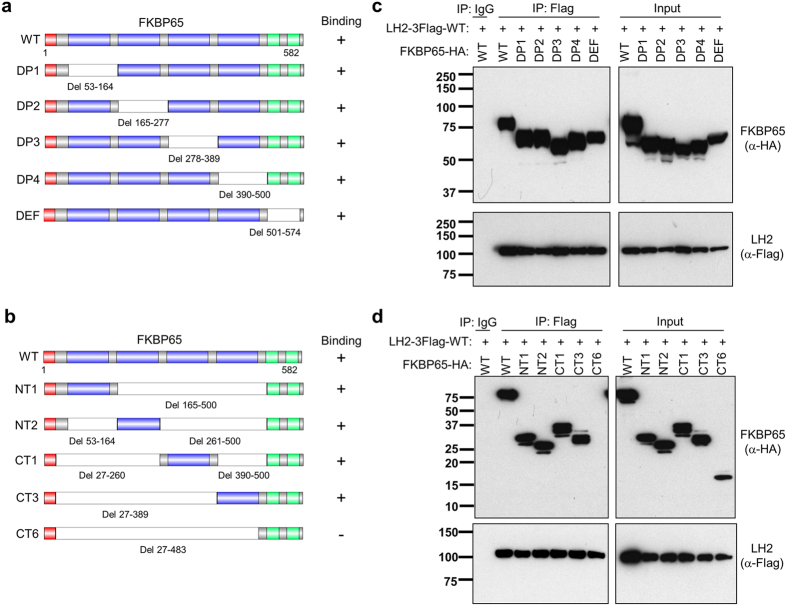
FKBP65 interacts with LH2 through its PPIase domains. (**a**, **b**) Schema of deletion mutants of FKBP65. Domains illustrated include signal peptide (red), PPIase (blue), and EF hand (green). Deleted regions are indicated in white. (**c**, **d**) Western blot analysis of 293 F cells transfected with the indicated plasmids. LH2 was immunoprecipitated with anti-Flag, and the immunprecipitates were subjected to Western blotting analysis.

To determine whether FKBP65 and LH2 are part of a common protein complex, we performed experiments on 293F cells co-transfected with tagged forms of LH2 and FKBP65, which showed that ectopic FKBP65 and LH2 co-immunoprecipitated from cell lysates (Fig. 3a) and co-localized in intact cells (Fig. 3b,c). As a measure of protein proximity in intact cells, we performed Gaussia luciferase complementation experiments^23^ using vectors that express LH2 fused to the N-terminus of Gaussia luciferase or FKBP65 fused to the C-terminus of Gaussia luciferase. Luciferase activity was 800-fold higher in co-transfected cells than in cells transfected with either vector alone (Fig. 3d), supporting the formation of a common protein complex. Protein complex formation was similar in 293F cells co-transfected with tagged forms of LH2 and wild-type or mutant (8FY) FKBP65 (Fig. 3e), arguing that PPIase activity is not required for protein complex formation. FKBP65 has four FKBP domains arranged in a linear extended structure followed by two EF hand domains^24^,^25^. Protein complex formation was maintained following deletion of single (Fig. 4a,c) or multiple (Fig. 4b,d) FKBP domains but not following deletion of all 4 FKBP domains (Fig. 4d), indicating that one FKBP domain is sufficient for FKBP65 to form a protein complex containing LH2.

## Discussion

In this study, by utilizing FKBP65-deficient MEFs as a platform, we demonstrated that loss of FKBP65 impairs LH2 function, resulting in a collagen cross-link switch from the Hyl^ald^ to the Lys^ald^-pathway, without affecting the quantity of total aldehydes involved in cross-links. The HLCC-to-LCC ratio was rescued in FKBP65-deficient murine embryonic fibroblasts by reconstitution with wild-type but not mutant FKBP65 that lacks intact PPIase domains. Furthermore, LH2 and FKBP65 were shown to be part of a common protein complex. While these findings support the conclusion that FKBP65 PPIase activity is required for LH2-driven collagen cross-linking activity, we were unable to generate intact recombinant FKBP65 protein to confirm that the mutant FKBP65 lacked PPIase activity but not chaperone activity, which is a limitation of the study.

The critical importance of collagen Lys hydroxylation in connective tissue physiology is well established^4^. By the late 1990s, three genes encoding isoforms of LH (LH1-3) were identified and partially characterized^26^,^27^. LH2’s function as a telopeptidyl LH was first proposed by Uzawa *et al*.^6^ and subsequently confirmed by several groups^7^,^8^,^28^,^29^. Though it is still not entirely clear whether LH2 also functions as a helical LH, such function is likely minimal as the defective LH2 function did not change the overall Lys hydroxylation in collagen (Fig. 1e). This notion is also supported by the observations by Bank *et al*.^15^.

Mutations in the *PLOD2* gene encoding LH2 cause Bruck syndrome type 2, in which the telopeptidyl Lys hydroxylation in bone type 1 collagen is lacking, leading to an abnormal cross-linking pattern^15^. This molecular phenotype also occurs in bone type 1 collagen by mutations in FKBP10; thus, it has been speculated that FKBP65 may interact with LH2 and regulate its function^30^,^31^. However, the actual interaction between FKBP65 and LH2 was not proven until very recently.

During the preparation of this manuscript, two groups independently reported that FKBP65 does indeed interact with LH2 by forming a protein complex^21^,^32^. One of these studies^21^ showed that FKBP65 interacts with LH2, but not LH1 or LH3, and is critical for the dimerization and function of LH2. This explains why helical Lys hydroxylation is not significantly affected in bone type 1 collagen in Bruck syndrome 1 and 2 as reported previously^15^,^30^,^33^ and in our study (Fig. 1d). In another study^32^, FKBP65 was shown to be part of a larger chaperone complex containing HSP47 and BiP, and was required to maintain LH2 protein levels. In our study, however, we found no differences in LH2 levels in FKBP10-null and –wild-type MEFs. This discrepancy may be explained by the use of fibroblasts from a distinct species (human versus murine) that contain a FKBP10 Gly278ArgfsX295 mutation, which leads to a truncated FKBP65 protein that is unstable but not completely lost. Moreover, our study provides important information by showing that FKBP65 regulates LH2-mediated collagen cross-linking and that FKBP65’s PPIase domains are critical for the integration of FKBP65 into a protein complex containing LH2.

It has been reported that LH2 is regulated at the transcriptional level by hypoxia-inducible factor 1α, TGF-β and several profibrotic cytokines, c-Myb, and microRNA-26a/b^12^,^13^,^34^,^35^,^36^,^37^,^38^. Our finding that LH2 protein levels were similar between *Fkbp10*^+/+^ and *Fkbp10*^−/−^ MEFs indicates that LH2 function is regulated post-translationally by FKBP65. In agreement with this finding, we showed for the first time that FKBP65 and LH2 are part of a common protein complex and that FKBP65, through its PPIase activity, controls the LH2-mediated collagen cross-link switch. In contrast, it has been reported that the PPIase activity of FKBP65 is not involved in the modulation of the coacervation and *in vitro* self-assembly of human tropoelastin^39^,^40^, indicating that FKBP65 regulates different biologic processes through different mechanisms. Recently, we reported that the PPIase cyclophilin B interacts with all LH1-3 and FKBP65, and that loss of cyclophilin B in mice leads to enhanced LH2 activity generating HLCCs in tendon type 1 collagen^41^. These together with the current findings strongly indicate an intricate interplay of these ER-resident PPIases with specific LH isoforms, thus, tightly controlling this important post-translational modification^42^.

Tissue-specific collagen cross-linking patterns have been well recognized for decades and are likely to be critical for the tissue-specific LH functions^2^,^43^,^44^. For instance, the specific and dynamic cross-linking pattern in bone/dentin type 1 collagen is important to regulate the process of collagen mineralization^45^,^46^,^47^,^48^. However, the tissue-specific cross-linking pattern cannot be explained solely by the respective LH gene expression^41^. Likely, these multifaceted post-translational control mechanisms of LH function are, in part, an important contributor to the cell- and tissue-specific collagen cross-linking. Further studies are warranted to understand how different PPIases regulate the function of LH2.

LH2-mediated collagen cross-linking has been implicated in pathologies. *In vitro*, altered expression of LH2 leads to abnormal collagen fibrillogenesis and matrix mineralization^28^. In addition, high levels of LH2 are associated with fibrosis^37^,^49^ and cancer metastasis^11^,^12^,^13^. In lung cancer, our group has recently shown that LH2 switches LCCs to more stable HLCCs in tumor stroma, which results in increased stromal stiffness, enhanced tumor growth, and metastasis^11^, suggesting that LH2 is a potential therapeutic target for preventing tumor metastasis. However, small molecules that directly target LH2’s hydroxylase activity are not available. Our finding that the PPIase activity of FKBP65 is required for the LH2-mediated collagen cross-link switch provides an alternative approach for manipulating the function of LH2 by targeting the PPIase activity of FKBP65. Supporting the feasibility of such inhibitors, tacrolimus (originally designated FK506) and sirolimus (rapamycin), which bind directly to FKBP12, have been developed as immunosuppressive drugs to be given after allogeneic organ transplant^50^,^51^. However, FK506 had no detectable effect on collagen cross-link formation in MEFs (data not shown). Potentially underlying our finding that LH2-induced collagen cross-links were inhibited by genetic, but not pharmacologic, inhibition of FKBP65, the knockout completely inhibits FKBP65 PPIase activity, whereas FK506 treatment achieves only a partial (~25%) reduction^25^, arguing that more potent FKBP65 inhibitors are needed. Such inhibitors may complement currently available anti-stromagenesis strategies, such as inhibitors of lysyl oxidase and lysyl oxidase–like family members that have demonstrated anti-metastatic activity in preclinical models^52^.

## Materials and Methods

### Cell culture

All cells were grown in a humidified atmosphere with 5% CO_2_ at 37 °C. *Fkbp10*^+/+^ MEFs (H2 and H3) and *Fkbp10*^−/−^ MEFs (G7 and G8) were kindly provided by Dr. Brendan Lee and Caressa D. Lietman at Baylor College of Medicine, and cultured in Dulbecco’s modified Eagle’s medium supplemented with 10% fetal bovine serum. HCC1438 cells were kindly provided by Dr. John Heymach in our department. 293T and 293F cells were obtained from ATCC and Thermo Fisher Scientific. These cells were cultured in a 1:1 mixture of Dulbecco’s modified Eagle’s medium and Ham’s F12 medium supplemented with 10% fetal bovine serum.

### Plasmids

To create a pLVX-Puro2 plasmid, we modified a pLVX-Puro plasmid (Clontech Laboratories, Mountain View, CA) by adding the SbfI and NotI cutting sites after the XbaI site. C-terminal 3Flag-tagged wild-type (WT) LH2 cDNAs were cloned into the XbaI and NotI sites on pLVX-Puro2 to generate an LH2-3Flag-WT expression plasmid. To create a pEF-bsr plasmid for the rescue experiment, we modified the pLVX-Puro plasmid by replacing the cytomegalovirus promoter with the EF1α promoter, replacing the puromycin-resistant gene with the blasticidin-resistant gene, and adding the SbfI and NotI cutting sites after the XbaI site. C-terminal hemagglutinin (HA)–tagged WT FKBP65 cDNA was cloned into XbaI and NotI sites on pEF-bsr to generate an FKBP65 expression plasmid. The deletion mutants of FKBP65 were generated by overlap polymerase chain reaction and cloned into the pEF-bsr plasmids. The construct schemas illustrated in Fig. 3 were generated using the DOG 2.0 tool for visualization of protein domain structures^53^. FKBP65-8FY cDNA was synthesized by GenScript (Piscataway, NJ) and cloned into the XbaI and NotI sites on pEF-bsr to generate an FKBP65-8FY expression plasmid. The N-terminus (G1, amino acids 1–93) and the C-terminus (G2, amino acids 94–169) of Gaussia luciferase were fused to the C-terminus of WT LH2 and the C-terminus of FKBP65, respectively with the polypeptide linker (Gly.Gly.Gly.Gly.Ser)_2_ and cloned into the XbaI and NotI sites on pLVX-Puro2 to generate LH2-G1 and FKBP65-G2 expression plasmids. Amino acid sequences are shown in Supplementary Table 1.

### Generation of stable cell lines

To restore FKBP65 expression, *Fkbp10*^−/−^ G7 MEFs were infected with lentiviruses carrying vector control, FKBP65-WT, or FKBP65-8FY. For knock down of FKBP65, the CHO cells were infected with lentiviruses carrying the scramble control or the FKBP65 shRNA. All lentiviruses were packaged in 293 T cells using pMD2.G (Addgene plasmid #12259) and psPAX2 plasmids (Addgene plasmid #12260). After 48 h, the MEFs cells were selected with blasticidin (10 μg/ml) and the CHO cells were selected with puromycin (10 μg/ml) for 2 weeks to generate stably transfected cells. The pMD2.G and psPAX2 plasmids were gifts from Dr. Didier Trono.

### Protein-fragment complementation assay

Plasmids with the LH2 and FKBP65 fusions were co-transfected at a 1:1 ratio into 293F cells plated on 24-well plates using the FuGENE 6 transfection reagent (Promega, Madison, WI). After 48 h, the cells were washed with phosphate-buffered saline (PBS), 400 μl of passive lysis buffer (Promega) was added to each well, and the plate was shaken for 15 min at room temperature. Then, 100 μl of the lysate was mixed with 100 μl of native coelenterazine (NanoLight Technology, Pinetop, AZ) stock solution. Native coelenterazine was used at a final concentration of 20 μM. The signal intensities of the reaction were read using a Synergy 2 microplate reader (BioTek Instruments, Inc., Winooski, VT).

### Western blotting

Western blotting was performed as described previously^11^. Briefly, cells were washed with PBS and lysed with cell lysis buffer (Cell Signaling Technology, Danvers, MA) to extract total proteins. Cell lysates were separated by sodium dodecyl sulfate polyacrylamide gel electrophoresis, transferred onto nitrocellulose transfer membrane (GE Healthcare Bio-Sciences, Pittsburgh, PA), and then incubated with primary antibodies and horseradish peroxidase–conjugated secondary antibodies (GE Healthcare Bio-Sciences). Protein bands were visualized with Pierce ECL Western blotting substrate (Thermo Fisher Scientific, Waltham, MA). The antibodies used were LH2 (#21214-1-AP) and FKBP65 (#12172-1-AP) from Proteintech Group, Rosemont, IL; actin (#A2066) and Flag (#F1804) from Sigma-Aldrich, St. Louis, MO; and HA (#3724) from Cell Signaling Technology.

### Immunoprecipitation

Immunoprecipitation experiments were performed as described previously^41^. Briefly, 293F cells were transfected with LH2-3Flag and FKBP65-HA (WT and deletion mutant) plasmids using FuGENE 6 transfection reagent (Promega). At 48 h after transfection, cells were lysed with cell lysis buffer (Cell Signaling Technology), and the total cell lysates were precleared with PBS with protein G agarose. The lysates were incubated with the Flag or HA antibody on a rotator at 4 °C overnight, and protein G beads were added to pull down the antibody-protein complexes. The beads were washed with PBS supplemented with phenylmethylsulfonyl fluoride five times, and the protein complexes were resolved from the beads by adding 1 × sodium dodecyl sulfate loading buffer and boiling for 5 min. The protein samples were subjected to Western blotting analysis to detect the presence of proteins with the Flag and HA antibodies.

### Immunofluorescence staining

HCC1438 cells were cultured on glass coverslips and transfected with LH2-3Flag and FKBP65-HA expression plasmids. After 48 h, the cells were fixed with formaldehyde, permeabilized, and incubated with HA and Flag antibodies followed by fluorescein isothiocyanate–conjugated secondary antibody and donkey anti-rabbit Alexa Fluor 594 (Thermo Fisher Scientific). Stained cells were visualized under a TCS SP8 confocal microscope (Leica Microsystems, Wetzlar, Germany) using a 63 × 1.4 numerical aperture oil objective. High-resolution images were acquired as described elsewhere^54^. Images were deconvolved, and colocalization was analyzed using Huygens Professional software (Scientific Volume Imaging B.V., Hilversum, The Netherlands).

### Collagen analysis

For the collagen cross-link analysis, the MEFs and their transfectants (n = 3/group) were cultured for 2 weeks supplemented with ascorbic acid at 100 μg/ml. FK506 treatment (0.5 or 1.0 μM) of the wild-type MEFs was initiated at time of confluence and continued for 14 days. The cell/matrix layer was first washed with cold PBS, scraped, and collected by centrifugation at 10,000 rpm for 30 min. The residues were further washed with cold PBS and cold distilled water by centrifugation, lyophilized, and weighed. Aliquots were reduced with standardized NaB^3^H_4_ and hydrolyzed with 6 N HCl. The hydrolysates were then subjected to amino acid and cross-link analyses as described previously^55^. The extent of Lys hydroxylation of collagen was calculated as Hyl/hydroxyproline (Hyp) X300 based on the value of 300 residues of Hyp per collagen. Upon reduction, the reducible cross-links, i.e. deH-DHLNL/ketoamine (Hyl^ald^ × Hyl), deH-HLNL/ketoamine (Hyl^ald^ × Lys or Lys^ald^ × Hyl) and deH-HHMD (Lys^ald^ x Lys^ald^ × Histidine × Hyl), were reduced to DHLNL, HLNL, and HHMD, respectively. Hereafter, the terms DHLNL, HLNL, and HHMD will be used for both the unreduced and the reduced forms. These reducible cross-links were identified and measured as their reduced forms. The mature trivalent cross-links, Pyr and d-Pyr, were simultaneously analyzed by their fluorescence. Another trivalent, non-reducible cross-link, pyrrole, was not analyzed due to its inherent lability during acid hydrolysis. All cross-links were quantified as moles per mole collagen (mole/mole collagen) based on the value of 300 residues of Hyp per collagen molecule. The total number of aldehydes involved in the cross-links analyzed was calculated as a sum of DHLNL, HLNL, 2 × Pyr, 2 × d-Pyr, and 2 × HHMD^4^.

### Statistical analysis

The data were analyzed using student’s t-test for significance in GraphPad Prism 6 software. The difference was considered significant at P < 0.05 (two-tailed).

## Additional Information

**How to cite this article:** Chen, Y. *et al*. FKBP65-dependent peptidyl-prolyl isomerase activity potentiates the lysyl hydroxylase 2-driven collagen cross-link switch. *Sci. Rep.* **7**, 46021; doi: 10.1038/srep46021 (2017).

**Publisher's note:** Springer Nature remains neutral with regard to jurisdictional claims in published maps and institutional affiliations.

## Supplementary Material

Supplementary Information
