# Supplementary material for: FKBP65-dependent peptidyl-prolyl isomerase activity potentiates the lysyl hydroxylase 2-driven collagen cross-link switch
Source: Sci Rep. 2017 Apr 5;7:46021. doi: 10.1038/srep46021 (PMC5380960; doi:10.1038/srep46021)
Supplement: Supplementary Information [file srep46021-s1.pdf]

FKBP65-dependent peptidyl-prolyl isomerase activity potentiates the lysyl hydroxylase 2-driven collagen cross-link switch

Yulong Chen[1], Masahiko Terajima[2], Priyam Banerjee[1], Houfu Guo[1], Xin Liu[1], Jiang Yu[1], Mitsuo Yamauchi[2,*] and Jonathan M. Kurie[1,*]

Supplementary Figure 1.

Supplementary Table 1.

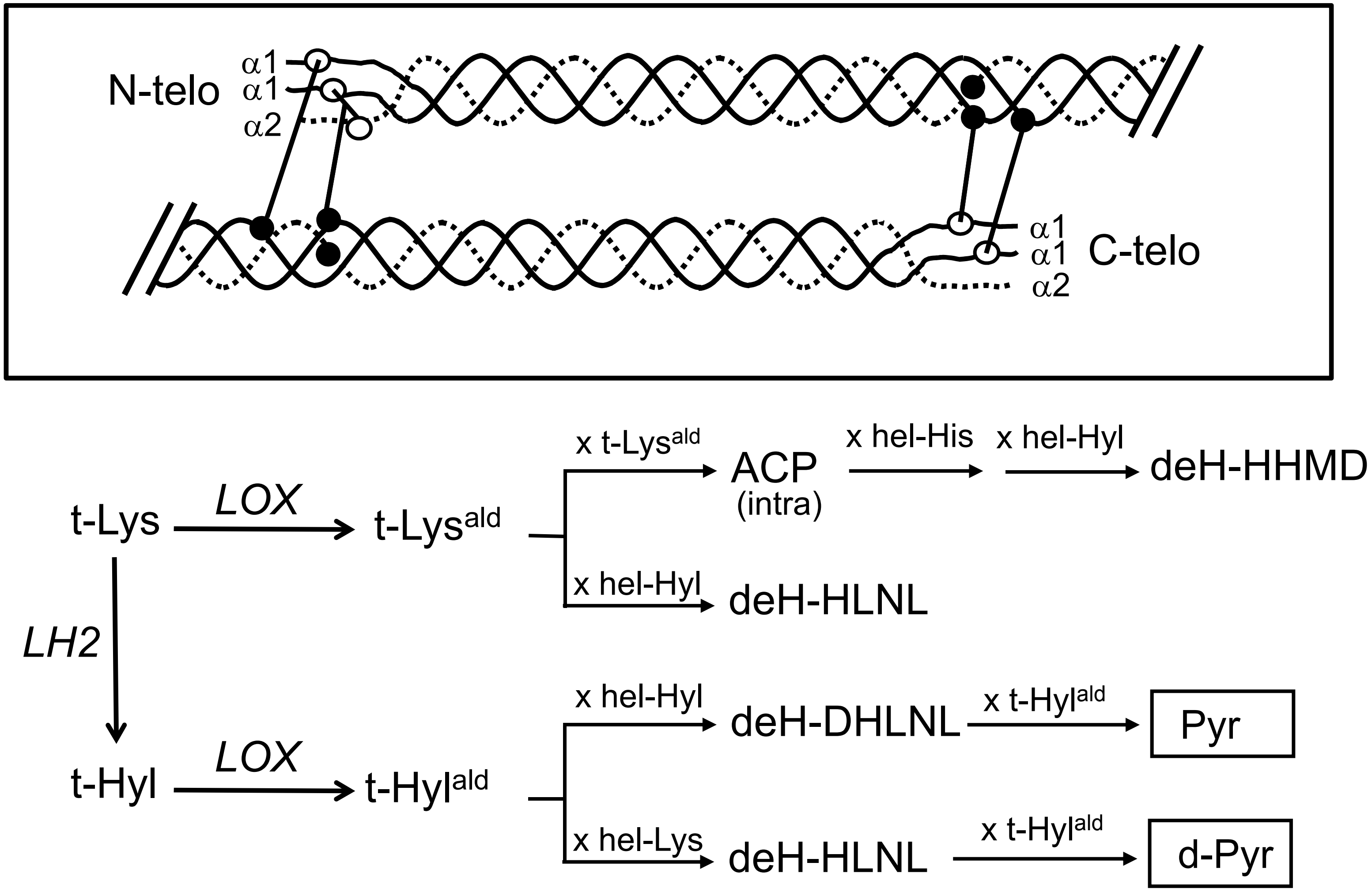

Supplementary Figure 1. The upper panel (boxed illustration) shows the cross-linking sites of type I collagen. Black lines within and between the molecules indicate examples of the intra- and inter-molecular cross-linkages. Numbers in parentheses indicate the residue numbers of the telopeptidyl aldehydes (open circles) and the helical lysine or hydroxylysine residues (closed circles) involved in cross-linking. C: carboxy-terminus, N: amino-terminus, telo: telopeptide. Solid line: a1 chain, dotted line: a2 chain
The lower panel summarizes collagen cross-linking pathways showing the cross-links analyzed in this study. The boxed cross-link compounds are non-reducible cross-links. t: telopeptidyl, hel: helical, Lys: lysine, Hyl: hydroxylysine, ald: aldehyde, His: histidine, LH2: Lysyl hydroxylase 2, LOX: Lysyl oxidase, ACP: Aldol condensation product (*Intra*molecular cross-link), deH : dehydro, HLNL: hydroxylysinonorleucine, DHLNL: dihydroxylysinonorleucine, HHMD: histidinohydroxymerodesmosine, HHL : histidinohydroxylysinonorleucine, Pyr : pyridinoline, d-: deoxy (Modified from Yamauchi and Sricholpech Essays Biochem 2012)

Supplementary Table 1. Amino acid sequences of constructs used.

LH2-3Flag:<br>MGGCTVKPQLLLLALVLHPWNPCLGADSEKPSSIPTDKLLVITVATKESDGFHRFMQSAKYFNYTVKVLG<br>QGEEWRGGDGINSIGGGQKVRLMKEVMEHYADQDDLVVMFTECFDVIFAGGPEEVLKKFQKANHKVVFAA<br>DGILWPDKRLADKYPVVHIGKRYLNSGGFIGYAPYVNRIVQQWNLQDNDDDQLFYTKVYIDPLKREAINI<br>TLDHKCKIFQTLNGAVDEVVLKFENGKARAKNTFYETLPVAINGNGPTKILLNYFGNYVPNSWTQDNGCT<br>LCEFDTVDLSAVDVHPNVSIGVFIEQPTPFLPRFLDILLTLDYPKEALKLFIHNKEVYHEKDIKVFFDKA<br>KHEIKTIKIVGPEENLSQAEARNMGMDFCRQDEKCDYYFSVDADVVLTNPRTLKILIEQNRKIIAPLVTR<br>HGKLWSNFWGALSPDGYYARSEDYVDIVQGNRVGVWNVPYMANVYLIKGKTLRSEMNERNYFVRDKLDPD<br>MALCRNAREMTLQREKDSPTPETFQMLSPPKGVFMYISNRHEFGRLLSTANYNTSHYNNDLWQIFENPVD<br>WKEKYINRDYSKIFTENIVEQPCPDVFWFPIFSEKACDELVEEMEHYGKWSGGKHHDSRISGGYENVPTD<br>DIHMKQVDLENVWLHFIREFIAPVTLKVFAGYYTKGFALLNFVVKYSPERQRSLRPHHDASTFTINIALN<br>NVGEDFQGGGCKFLRYNCSIESPRKGWSFMHPGRLTHLHEGLPVKNGTRYIAVSFIDPDYKDDDDKDYKD<br>DDDKDYKDDDDK*
LH2-G1:<br>MGGCTVKPQLLLLALVLHPWNPCLGADSEKPSSIPTDKLLVITVATKESDGFHRFMQSAKYFNYTVKVLG<br>QGEEWRGGDGINSIGGGQKVRLMKEVMEHYADQDDLVVMFTECFDVIFAGGPEEVLKKFQKANHKVVFAA<br>DGILWPDKRLADKYPVVHIGKRYLNSGGFIGYAPYVNRIVQQWNLQDNDDDQLFYTKVYIDPLKREAINI<br>TLDHKCKIFQTLNGAVDEVVLKFENGKARAKNTFYETLPVAINGNGPTKILLNYFGNYVPNSWTQDNGCT<br>LCEFDTVDLSAVDVHPNVSIGVFIEQPTPFLPRFLDILLTLDYPKEALKLFIHNKEVYHEKDIKVFFDKA<br>KHEIKTIKIVGPEENLSQAEARNMGMDFCRQDEKCDYYFSVDADVVLTNPRTLKILIEQNRKIIAPLVTR<br>HGKLWSNFWGALSPDGYYARSEDYVDIVQGNRVGVWNVPYMANVYLIKGKTLRSEMNERNYFVRDKLDPD<br>MALCRNAREMTLQREKDSPTPETFQMLSPPKGVFMYISNRHEFGRLLSTANYNTSHYNNDLWQIFENPVD<br>WKEKYINRDYSKIFTENIVEQPCPDVFWFPIFSEKACDELVEEMEHYGKWSGGKHHDSRISGGYENVPTD<br>DIHMKQVDLENVWLHFIREFIAPVTLKVFAGYYTKGFALLNFVVKYSPERQRSLRPHHDASTFTINIALN<br>NVGEDFQGGGCKFLRYNCSIESPRKGWSFMHPGRLTHLHEGLPVKNGTRYIAVSFIDPGGGGSGGGGSMK<br>PTENNEDFNIVAVASNFATTDLDADRGKLPGKKLPLEVLKEMEANARKAGCTRGCLICLSHIKCTPKMKK<br>FIPGRCHTYEGDKESAQGGIG*
FKBP65-WT (FKBP10-HA):<br>MFPAGPPSHSLLRLPLLQLLLVVQAVGRGLGRASPAGGPLEDVVIERYHIPRACPREVQMGDFVRYHYN<br>GTFEDGKKFDSSYDRNTLVAIVVGVGRLITGMDRGLMGMCVNERRRLIVPPHLGYGSIGLAGLIPPDATL<br>YFDVVLLDVWNKEDTVQVSTLLRPPHCPRMVQDGDFVRYHYNGTLLDGTSFDTSYSKGGTYDTYVGSGWL<br>IKGMDQGLLGMCPGERRKIIIPPFLAYGEKGYGTVIPPQASLVFHVLLIDVHNPKDAVQLETLELPPGCV<br>RRAGAGDFMRYHYNGSLMDGTLFDSSYSRNHTYNTYIGQGYIIPGMDQGLQGACMGERRRITIPPHLAYG<br>ENGTGDKIPGSAVLIFNVHVIDFHNPADVVEIRTLSRPSETCNETTKLGDFVRYHYNCSLLDGTQLFTSH<br>DYGAPQEATLGANKVIEGLDTGLQGMCVGERRQLIVPPHLAHGESGARGVPGSAVLLFEVELVSREDGLP<br>TGYLFVWHKDPPANLFEDMDLNKDGEVPPEEFSTFIKAQVSEGKGRLMPGQDPEKTIGDMFQNQDRNQDG<br>KITVDELKLKSDEDEERVHEELCYPYDVPDYASLHEEL*
FKBP65-DP1:<br>MFPAGPPSHSLLRLPLLQLLLVVQAVGRGLGRASPAGGPLEDVVIERYHIPPHCPRMVQDGDFVRYHYN<br>GTLLDGTSFDTSYSKGGTYDTYVGSGWLIKGMDQGLLGMCPGERRKIIIPPFLAYGEKGYGTVIPPQASL<br>VFHVLLIDVHNPKDAVQLETLELPPGCVRRAGAGDFMRYHYNGSLMDGTLFDSSYSRNHTYNTYIGQGYI<br>IPGMDQGLQGACMGERRRITIPPHLAYGENGTGDKIPGSAVLIFNVHVIDFHNPADVVEIRTLSRPSETC<br>NETTKLGDFVRYHYNCSLLDGTQLFTSHDYGAPQEATLGANKVIEGLDTGLQGMCVGERRQLIVPPHLAH<br>GESGARGVPGSAVLLFEVELVSREDGLPTGYLFVWHKDPPANLFEDMDLNKDGEVPPEEFSTFIKAQVSE<br>GKGRLMPGQDPEKTIGDMFQNQDRNQDGKITVDELKLKSDEDEERVHEELCYPYDVPDYASLHEEL*
FKBP65-DP2:<br>MFPAGPPSHSLLRLPLLQLLLVVQAVGRGLGRASPAGGPLEDVVIERYHIPRACPREVQMGDFVRYHYN<br>GTFEDGKKFDSSYDRNTLVAIVVGVGRLITGMDRGLMGMCVNERRRLIVPPHLGYGSIGLAGLIPPDATL

YFDVVLLDVWNKEDTVQVSTLLRPGCVRRAGAGDFMRYHYNGSLMDGTLFDSSYSRNHTYNTYIGQGYII
PGMDQGLQGACMGERRRITIPPHLAYGENGTGDKIPGSAVLIFNVHVIDFHNPADVVEIRTLSRPSETCN
ETTKLGDFVRYHYNCSLLDGTQLFTSHDYGAPQEATLGANKVIEGLDTGLQGMCVGERRQLIVPPHLAHG
ESGARGVPGSAVLLFEVELVSREDGLPTGYLFVWHKDPPANLFEDMDLNKDGEVPPEEFSTFIKAQVSEG
KGRLMPGQDPEKTIGDMFQNQDRNQDGKITVDELKLKSDEDEERVHEELCYPYDVPDYASLHEEL*

FKBP65-DP3:
MFPAGPPSHSLLRLPLLQLLLLVVQAVGRGLGRASPAGGPLEDVVIERYHIPRACPREVQMGDFVRYHYN
GTFEDGKKFDSSYDRNTLVAIVVGVGRLITGMDRGLMGMCVNERRRLIVPPHLGYGSIGLAGLIPPDATL
YFDVVLLDVWNKEDTVQVSTLLRPPHCPRMVQDGDFVRYHYNGTLLDGTSFDTSYSKGGTYDTYVGSGWL
IKGMDQGLLGMCPGERRKIIIPPFLAYGEKGYGTVIPPQASLVFHVLLIDVHNPKDAVQLETLELPPETC
NETTKLGDFVRYHYNCSLLDGTQLFTSHDYGAPQEATLGANKVIEGLDTGLQGMCVGERRQLIVPPHLAH
GESGARGVPGSAVLLFEVELVSREDGLPTGYLFVWHKDPPANLFEDMDLNKDGEVPPEEFSTFIKAQVSE
GKGRLMPGQDPEKTIGDMFQNQDRNQDGKITVDELKLKSDEDEERVHEELCYPYDVPDYASLHEEL*

FKBP65-DP4:
MFPAGPPSHSLLRLPLLQLLLLVVQAVGRGLGRASPAGGPLEDVVIERYHIPRACPREVQMGDFVRYHYN
GTFEDGKKFDSSYDRNTLVAIVVGVGRLITGMDRGLMGMCVNERRRLIVPPHLGYGSIGLAGLIPPDATL
YFDVVLLDVWNKEDTVQVSTLLRPPHCPRMVQDGDFVRYHYNGTLLDGTSFDTSYSKGGTYDTYVGSGWL
IKGMDQGLLGMCPGERRKIIIPPFLAYGEKGYGTVIPPQASLVFHVLLIDVHNPKDAVQLETLELPPGCV
RRAGAGDFMRYHYNGSLMDGTLFDSSYSRNHTYNTYIGQGYIIPGMDQGLQGACMGERRRITIPPHLAYG
ENGTGDKIPGSAVLIFNVHVIDFHNPADVVEIRTLSRPSPPANLFEDMDLNKDGEVPPEEFSTFIKAQVS
EGKGRLMPGQDPEKTIGDMFQNQDRNQDGKITVDELKLKSDEDEERVHEELCYPYDVPDYASLHEEL*

FKBP65-DEF:
MFPAGPPSHSLLRLPLLQLLLLVVQAVGRGLGRASPAGGPLEDVVIERYHIPRACPREVQMGDFVRYHYN
GTFEDGKKFDSSYDRNTLVAIVVGVGRLITGMDRGLMGMCVNERRRLIVPPHLGYGSIGLAGLIPPDATL
YFDVVLLDVWNKEDTVQVSTLLRPPHCPRMVQDGDFVRYHYNGTLLDGTSFDTSYSKGGTYDTYVGSGWL
IKGMDQGLLGMCPGERRKIIIPPFLAYGEKGYGTVIPPQASLVFHVLLIDVHNPKDAVQLETLELPPGCV
RRAGAGDFMRYHYNGSLMDGTLFDSSYSRNHTYNTYIGQGYIIPGMDQGLQGACMGERRRITIPPHLAYG
ENGTGDKIPGSAVLIFNVHVIDFHNPADVVEIRTLSRPSETCNETTKLGDFVRYHYNCSLLDGTQLFTSH
DYGAPQEATLGANKVIEGLDTGLQGMCVGERRQLIVPPHLAHGESGARGVPGSAVLLFEVELVSREDGLP
TGYLFVWHKDEERVHEELCYPYDVPDYASLHEEL*

FKBP65-NT1:
MFPAGPPSHSLLRLPLLQLLLLVVQAVGRGLGRASPAGGPLEDVVIERYHIPRACPREVQMGDFVRYHYN
GTFEDGKKFDSSYDRNTLVAIVVGVGRLITGMDRGLMGMCVNERRRLIVPPHLGYGSIGLAGLIPPDATL
YFDVVLLDVWNKEDTVQVSTLLRPPPANLFEDMDLNKDGEVPPEEFSTFIKAQVSEGKGRLMPGQDPEKT
IGDMFQNQDRNQDGKITVDELKLKSDEDEERVHEELCYPYDVPDYASLHEEL*

FKBP65-NT2:
MFPAGPPSHSLLRLPLLQLLLLVVQAVGRGLGRASPAGGPLEDVVIERYHIPPHCPRMVQDGDFVRYHYN
GTLLDGTSFDTSYSKGGTYDTYVGSGWLIKGMDQGLLGMCPGERRKIIIPPFLAYGEKGYGTVIPPQASL
VFHVLLIDPPANLFEDMDLNKDGEVPPEEFSTFIKAQVSEGKGRLMPGQDPEKTIGDMFQNQDRNQDGKI
TVDELKLKSDEDEERVHEELCYPYDVPDYASLHEEL*

FKBP65-CT1:
MFPAGPPSHSLLRLPLLQLLLLVVQAVHNPKDAVQLETLELPPGCVRRAGAGDFMRYHYNGSLMDGTLFD
SSYSRNHTYNTYIGQGYIIPGMDQGLQGACMGERRRITIPPHLAYGENGTGDKIPGSAVLIFNVHVIDFH
NPADVVEIRTLSRPSPPANLFEDMDLNKDGEVPPEEFSTFIKAQVSEGKGRLMPGQDPEKTIGDMFQNQD
RNQDGKITVDELKLKSDEDEERVHEELCYPYDVPDYASLHEEL*

FKBP65-CT3:
MFPAGPPSHSLLRLPLLQLLLLVVQAETCNETTKLGDFVRYHYNCSLLDGTQLFTSHDYGAPQEATLGAN
KVIEGLDTGLQGMCVGERRQLIVPPHLAHGESGARGVPGSAVLLFEVELVSREDGLPTGYLFVWHKDPPA

NLFEDMDLNKDGEVPPEEFSTFIKAQVSEGKGRLMPGQDPEKTIGDMFQNQDRNQDGKITVDELKLKSDE<br>DEERVHEELCYPYDVPDYASLHEEL*
FKBP65-CT6:<br>MFPAGPPSHSLLRLPLLQLLLLVVQASREDGLPTGYLFVWHKDPPANLFEDMDLNKDGEVPPEEFSTFIK<br>AQVSEGKGRLMPGQDPEKTIGDMFQNQDRNQDGKITVDELKLKSDEDEERVHEELCYPYDVPDYASLHEE<br>L*
FKBP65-8FY:<br>MFPAGPPSHSLLRLPLLQLLLLVVQAVGRGLGRASPAGGPLEDVVIERYHIPRACPREVQMGDFVRYHYN<br>GTFEDGKKYDSSYDRNTLVAIVVGVGRLITGMDRGLMGMCVNERRRLIVPPHLGYGSIGLAGLIPPDATL<br>YYDVVLLDVWNKEDTVQVSTLLRPPHCPRMVQDGDFVRYHYNGTLLDGTSYDTSYSKGGTYDTYVGSGWL<br>IKGMDQGLLGMCPGERRKIIIPPFLAYGEKGYGTVIPPQASLVYHVLLIDVHNPKDAVQLETLELPPGCV<br>RRAGAGDFMRYHYNGSLMDGTLYDSSYSRNHTYNTYIGQGYIIPGMDQGLQGACMGERRRITIPPHLAYG<br>ENGTGDKIPGSAVLIYNVHVIDFHNPADVVEIRTLSRPSETCNETTKLGDFVRYHYNCSLLDGTQLYTSH<br>DYGAPQEATLGANKVIEGLDTGLQGMCVGERRQLIVPPHLAHGESGARGVPGSAVLLYEVELVSREDGLP<br>TGYLFVWHKDPPANLFEDMDLNKDGEVPPEEFSTFIKAQVSEGKGRLMPGQDPEKTIGDMFQNQDRNQDG<br>KITVDELKLKSDEDEERVHEELCYPYDVPDYASLHEEL*
FKBP65-G2:<br>MFPAGPPSHSLLRLPLLQLLLLVVQAVGRGLGRASPAGGPLEDVVIERYHIPRACPREVQMGDFVRYHYN<br>GTFEDGKKFDSSYDRNTLVAIVVGVGRLITGMDRGLMGMCVNERRRLIVPPHLGYGSIGLAGLIPPDATL<br>YFDVVLLDVWNKEDTVQVSTLLRPPHCPRMVQDGDFVRYHYNGTLLDGTSFDTSYSKGGTYDTYVGSGWL<br>IKGMDQGLLGMCPGERRKIIIPPFLAYGEKGYGTVIPPQASLVFHVLLIDVHNPKDAVQLETLELPPGCV<br>RRAGAGDFMRYHYNGSLMDGTLFDSSYSRNHTYNTYIGQGYIIPGMDQGLQGACMGERRRITIPPHLAYG<br>ENGTGDKIPGSAVLIFNVHVIDFHNPADVVEIRTLSRPSETCNETTKLGDFVRYHYNCSLLDGTQLFTSH<br>DYGAPQEATLGANKVIEGLDTGLQGMCVGERRQLIVPPHLAHGESGARGVPGSAVLLFEVELVSREDGLP<br>TGYLFVWHKDPPANLFEDMDLNKDGEVPPEEFSTFIKAQVSEGKGRLMPGQDPEKTIGDMFQNQDRNQDG<br>KITVDELKLKSDEDEERVHEELGGGGSGGGGSEAIVDIPEIPGFKDLEPMEQFIAQVDLCVDCTTGCLKG<br>LANVQCSDLLKKWLPQRCATFASKIQGQVDKIKGAGGD*

Signal peptide; Endoplasmic reticulum retention peptide; 3x-Flag; HA; Linker peptide; N-terminal (G1) fragment of G-luciferase; C-terminal (G2) fragment of G-luciferase.
